# The resolution revolution in cryoEM requires high-quality sample preparation: a rapid pipeline to a high-resolution map of yeast fatty acid synthase

**DOI:** 10.1107/S2052252519017366

**Published:** 2020-01-25

**Authors:** Mirko Joppe, Edoardo D’Imprima, Nina Salustros, Karthik S. Paithankar, Janet Vonck, Martin Grininger, Werner Kühlbrandt

**Affiliations:** a Institute of Organic Chemistry and Chemical Biology, Buchmann Institute for Molecular Life Sciences, Goethe University Frankfurt, Max-von-Laue-Strasse 15, 60438 Frankfurt am Main, Germany; b Department of Structural Biology, Max Planck Institute of Biophysics, Max-von-Laue Strasse 3, 60438 Frankfurt am Main, Germany

**Keywords:** purification of protein complexes, 3D reconstruction and image processing, single-particle cryoEM, cryo-electron microscopy, macromolecular machines, protein structure, yeast fatty acid synthase

## Abstract

Single-particle electron cryo-microscopy (cryoEM) has undergone a ‘resolution revolution’ that makes it possible to characterize megadalton (MDa) complexes at atomic resolution without crystals. To fully exploit the new opportunities in molecular microscopy, new procedures for the cloning, expression and purification of macromolecular complexes need to be explored. Macromolecular assemblies are often unstable, and invasive construct design or inadequate purification conditions and sample-preparation methods can result in disassembly or denaturation. The structure of the 2.6 MDa yeast fatty acid synthase (FAS) has been studied by electron microscopy since the 1960s. Here, a new, streamlined protocol for the rapid production of purified yeast FAS for structure determination by high-resolution cryoEM is reported. Together with a companion protocol for preparing cryoEM specimens on a hydrophilized graphene layer, the new protocol yielded a 3.1 Å resolution map of yeast FAS from 15 000 automatically picked particles within a day. The high map quality enabled a complete atomic model of an intact fungal FAS to be built.

## Abstract 2

Optimized protocols for protein production and purification as well as cryoEM grid preparation allow the reconstruction of a 3D map of yeast fatty acid synthase (FAS) at ∼3 Å resolution from a comparatively small number of particle images within a short time. The first complete model of yeast FAS is presented.

## 1. Introduction   

Recent developments in single-particle cryoEM make it possible to determine the structures of large macromolecular complexes that are not available in sufficiently large amounts or that resist crystallization. In cryoEM, individual complexes are imaged in a thin layer of vitrified buffer (McDowall *et al.*, 1983 ▸). With the recently developed direct electron detectors (McMullan *et al.*, 2009 ▸) and image-processing packages (Cheng *et al.*, 2015 ▸), cryoEM has become increasingly powerful, and it is now the method of choice for determining the structures of large macromolecular assemblies at high resolution. New image-processing algorithms can deal with sample heterogeneity, and analyzing this heterogeneity often provides direct insights into molecular mechanisms (Zivanov *et al.*, 2018 ▸; Punjani *et al.*, 2017 ▸; Grant *et al.*, 2018 ▸; Murphy *et al.*, 2019 ▸). It is no longer uncommon for cryoEM to achieve resolutions of 3 Å or better. To date, more than 200 cryoEM structures in this high resolution range have been deposited in the Electron Microscopy Data Bank (EMDB; http://emdatabank.org/). In the same way as X-ray structures, the new high-resolution cryoEM structures serve as a base for designing inhibitors or mutants and for analyzing biomolecular interfaces.

The yeast fatty acid synthase (FAS) was one of the first protein complexes to be analyzed in structural biology. Since the mid 1960s, dozens of studies have described the overall structure of the 2.6 MDa complex and its individual domains (Lynen *et al.*, 1980 ▸; Maier *et al.*, 2010 ▸). Although today the mechanism of modular fatty-acid synthesis is well understood, FAS remains an important target for structural and functional studies. Yeast FAS is a prime example for revealing co-translational subunit association as a mechanism in the assembly of eukaryotic proteins (Shiber *et al.*, 2018 ▸; Fischer *et al.*, 2020 ▸). It is also critical for the production of fatty acids in microbes as a platform for chemical synthesis (Gajewski, Pavlovic *et al.*, 2017 ▸; Zhu *et al.*, 2017 ▸). So far, FAS has been purified from natural sources (Lynen, 1969 ▸), but it is now becoming increasingly important to develop mutants, which are expressed in recombinant systems (Maier, 2017 ▸; Heil *et al.*, 2019 ▸). At the same time, requirements for high-quality protein preparations for structural studies are becoming more stringent.

To meet these requirements, we developed a new protocol for the rapid preparation of recombinantly expressed yeast FAS. Our protocol includes vector-based expression under the native promoter, non-invasive affinity chromatography and strict monitoring of protein integrity. Taking advantage of a companion protocol that prevents protein denaturation at the air–water interface (D’Imprima *et al.*, 2019 ▸), we show that we can reconstruct a 3D map of yeast FAS at ∼3 Å resolution from a comparatively small number of particle images within a short time. The same approach can now be used for other macromolecular assemblies.

## 2. Materials and methods   

### 2.1. Strain cultivation and protein purification   

Yeast cultures were grown and FAS was purified as reported previously (Gajewski, Pavlovic *et al.*, 2017 ▸; D’Imprima *et al.*, 2019 ▸). Haploid FAS-deficient *Saccharomyces cerevisiae* cells were transfected with plasmids carrying FAS-encoding genes and grown in YPD medium. After bead disruption and differential centrifugation, the soluble components were purified by Strep-Tactin affinity chromatography followed by size-exclusion chromatography. The main peak was collected. During purification, FAS was kept in buffer P1 (100 m*M* sodium phosphate pH 6.5). Purification was monitored by SDS–PAGE.

### 2.2. Thermal shift assay (TSA) and activity assay   

**Figure 1:**
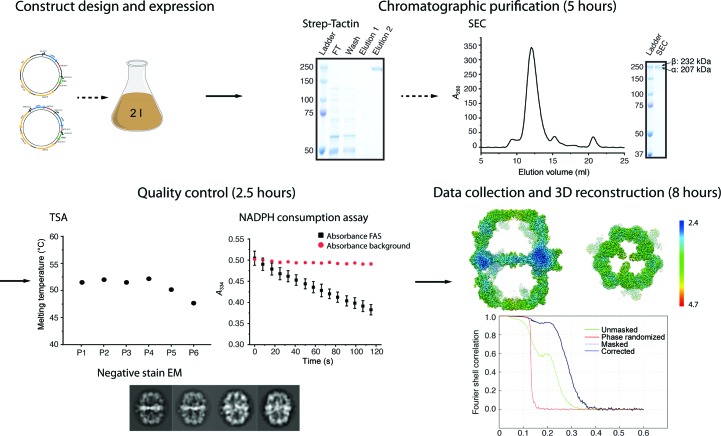
Structural analysis of yeast FAS. Yeast FAS was expressed overnight from pRS vector-encoded FAS1 and FAS2 genes. Gravity flow of the cleared lysate over a Strep-Tactin column and subsequent size-exclusion chromatography (SEC) delivered pure protein within 5 h. Protein quality was monitored by NADPH consumption, thermal shift assays (TSA) and negative-stain transmission EM within 2.5 h. Thermal stability was tested for a set of conditions (P1, 100 m*M* sodium phosphate pH 6.5; P2, 100 m*M* sodium phosphate pH 7.4; P3, 100 m*M* sodium phosphate pH 8; P4, 100 m*M* sodium phosphate, 100 m*M* NaCl pH 7.4; P5, 100 m*M* Tris–HCl pH 7.4; P6, distilled water). The activity of the preparation was 2310 ± 48 mU mg^−1^ and the error in the melting point varied by less than 0.5°C; both values were within technical replication. Protein integrity was assessed further by negative-stain EM and 2D single-particle image analysis (within 1.5 h). CryoEM images were collected in movie mode in 4.5 h. 20 000 particles were picked automatically, of which 15 000 were selected by 2D and 3D classification, to yield a map at 3.1 Å resolution in 3.5 h of image processing.

Buffers P1, P2 (100 m*M* sodium phosphate pH 7.4), P3 (100 m*M* sodium phosphate pH 8), P4 (100 m*M* sodium phosphate, 100 m*M* NaCl pH 7.4), P5 (100 m*M* Tris–HCl pH 7.4) and distilled water were used in thermal shift assays (see also Fig. 1 ▸). Briefly, 2 µl protein solution (0.9 mg ml^−1^) was mixed with 21 µl buffer and 2 µl 62.5× SYPRO Orange protein gel stain, and fluorescence was then measured from 5 to 95°C with a step of 0.5°C min^−1^ with the excitation wavelength set to 450–490 nm and the emission wavelength set to 560–580 nm. FAS activity was determined by tracing NADPH consumption at 334 nm as reported in Gajewski, Buelens *et al.* (2017 ▸) and adapted for plate-reader readout (120 µl scale containing 200 m*M* NaH_2_PO_4_/Na_2_HPO_4_ pH 7.3, 1.75 m*M* 1,4-dithiothreitol, 0.03 mg ml^−1^ BSA, 0.7 µg FAS, 500 µ*M* malonyl-CoA, 417 µ*M* acetyl-CoA and 250 µ*M* NADPH).

### 2.3. Negative-stain electron microscopy   

FAS was diluted to 0.05 mg ml^−1^ in purification buffer P1 and was negatively stained with 2%(*w*/*v*) sodium silicotungstate (Agar Scientific, Stansted, England). Specimens were prepared by applying a 3 µl droplet of protein solution to 300 mesh carbon-coated copper grids freshly glow-discharged at 15 mA for 45 s (Structure Probe Inc., West Chester, Pennsylvania, USA). The sample was incubated for 1 min before blotting with Whatman No. 1 filter paper (Sigma–Aldrich, Munich, Germany). Subsequently, two changes of 3 µl of stain were applied to the specimens for 15 s before blotting. Finally, the grids were left at room temperature to dry. Micrographs were recorded with an FEI Tecnai G2 Spirit (FEI Company, Hillsboro, Oregon, USA) operated at 120 kV on a Gatan Ultrascan 4000 CCD camera at a pixel size of 2.68 Å.

### 2.4. CryoEM grid preparation   

Specimen preparation was carried out as described by D’Imprima *et al.* (2019 ▸). Briefly, Quantifoil R1.2/1.3 grids (Quantifoil Micro Tools, Jena, Germany) were washed overnight in chloroform (Sigma–Aldrich, Munich, Germany). The grids were coated with a single layer of graphene (Graphenea, Cambridge, Massachusetts, USA) stored in a sandwich of polyethylene terephthalate (PET) support and a protective layer of poly(methyl methacrylate) (PMMA). Graphene pads (1 cm^2^) were floated onto Quantifoil grids in a water bath where they were released from the PET support. Subsequently, the water was drained and graphene was layered carefully onto the grids. To ensure good adherence of the graphene, the grids were annealed at 150°C for 30 min. The graphene-coated grids were then washed in pure acetone and 2-propanol for 1 h each to remove the PMMA film and the grids were dried under a nitrogen stream. Other than during annealing, the graphene-coated grids were kept under a nitrogen stream in order to minimize air contamination of the graphene. Finally, the grids were dipped into 5 µ*M* 1-pyrenecarboxylic acid (Sigma–Aldrich, Munich, Germany) dissolved in DMSO (Sigma–Aldrich, Munich, Germany) for 1 min, rinsed in one change of 2-propanol and ethanol, and dried under a nitrogen stream. For all grids, the graphene layer was deposited on the carbon side of the Quantifoils, whereas the protein sample was subsequently applied to the copper side.

### 2.5. Single-particle cryoEM   

3 µl FAS solution (0.3 mg ml^−1^), incubated with 1 m*M* NADPH and 1 m*M* malonyl-CoA for 5 min at room temperature, was applied to the graphene-coated Quantifoil grids. The grids were vitrified in a Vitrobot Mark IV plunge-freezer at 100% humidity and 10°C after blotting for 6–8 s. CryoEM images were collected with a Titan Krios (FEI Company, Hillsboro, Oregon, USA) electron microscope operating at 300 kV. Images were recorded automatically with *EPU* at a pixel size of 0.833 Å on a Falcon III EC direct electron detector (FEI Company, Hillsboro, Oregon, USA) operating in counting mode. A total of 792 dose-fractionated movies were recorded with a cumulative dose of ∼32 e^−^ Å^−2^. Image drift correction and dose weighting were performed using *MotionCor*2 (Zheng *et al.*, 2017 ▸) within the *RELION*-3 pipeline (Zivanov *et al.*, 2018 ▸). CTF determination was performed with *CTFFIND* 4.1.13 (Rohou & Grigorieff, 2015 ▸). From a data set of 19 981 particles picked automatically with *crYOLO* (Wagner *et al.*, 2019 ▸), 15 320 remained after 2D and 3D classification in *cryoSPARC* (Punjani *et al.*, 2017 ▸). The particles contributing to the best 3D class were subjected to homogeneous and non-uniform refinement in *cryoSPARC*, yielding a map at 3.1 Å resolution, as determined by the post-processing procedure in *RELION* (Chen *et al.*, 2013 ▸).

### 2.6. Model building   

The X-ray model of yeast FAS (PDB entry 3hmj; Johansson *et al.*, 2009 ▸) was docked into the cryoEM map with *USCF Chimera* (Pettersen *et al.*, 2004 ▸) and manually rebuilt and completed in *Coot* (Emsley *et al.*, 2010 ▸). The model was refined using *phenix.real_space_refinement* (Liebschner *et al.*, 2019 ▸) with geometry and secondary-structure restraints, followed by manual inspection and adjustments in *Coot*. The geometry of the model was validated by *MolProbity* (Chen *et al.*, 2010 ▸).

## 3. Results   

### 3.1. Developing a protocol for FAS purification   

Previous procedures for the preparation of yeast FAS from baker’s yeast followed a sequence of ammonium sulfate fractionation, chromatography on calcium phosphate gels, ultracentrifugation and hydroxyapatite chromatography (Lynen, 1969 ▸). An improved variant that included additional chromatographic steps was used for the 3.1 Å resolution X-ray structure of baker’s yeast FAS (Leibundgut *et al.*, 2007 ▸; Lomakin *et al.*, 2007 ▸). A significantly shorter protocol was based on the modification of yeast FAS with a His tag integrated into the FAS1 gene by homologous recombination, which enabled nickel-chelating chromatography as the first purification step (Johansson *et al.*, 2008 ▸).

We recently established a plasmid-based expression system suitable for expressing FAS-encoding genes in baker’s yeast deletion strains (D’Imprima *et al.*, 2019 ▸). Here, the FAS1 gene was tagged with a Strep-Tag at the C-terminus of subunit β (Schmidt & Skerra, 2007 ▸). Strep-Tactin affinity chromatography followed by size-exclusion chromatography (SEC) delivered pure protein within 5 h. The protein is pure as judged by SDS–PAGE and has a specific activity of 2100 ± 300 mU mg^−1^, which is in the range reported for the best previous preparations of fungal FASs (Kolodziej *et al.*, 1996 ▸; Fichtlscherer *et al.*, 2000 ▸; Wieland *et al.*, 1979 ▸; Fischer *et al.*, 2015 ▸). The standard deviation of the specific enzymatic activities of FAS from nine independent preparations indicates that the protocol delivers protein of a significantly better, more reproducible quality than previous protocols. The normalized standard deviation of specific enzymatic activities in our study was 0.14, whereas previously it was 0.52 (Lynen, 1969 ▸).

The C-terminus of the β subunit was selected for affinity tagging, because it is stably anchored in the MPT domain, which is itself stably integrated into the main protein body (Johansson *et al.*, 2009 ▸; Gipson *et al.*, 2010 ▸). The suitability of the C-terminus of the β subunit for modifications with peptides and proteins has also recently been demonstrated by others: a 3×FLAG-tag fusion aided in the purification of FAS for studying ACP-mediated substrate shuttling (Lou *et al.*, 2019 ▸), and the FAS co-translational assembly pathway protein (Shiber *et al.*, 2018 ▸) as well as the autophagic degradation of FAS (Shpilka *et al.*, 2015 ▸) were monitored using a GFP fusion construct. To keep as closely as possible to physiological conditions, we put the encoding sequence on single copy number centromeric pRS shuttle vectors of types pRS313 and pRS315 (Sikorski & Hieter, 1989 ▸; Gajewski, Pavlovic *et al.*, 2017 ▸). Expression yielded 1.4 ± 0.4 mg yeast FAS from a 2 l culture within 5 h. The plasmid-encoded expression system enables rapid and economical mutagenesis and tolerates lethal phenotypes induced by FAS mutations when external fatty acids are supplied (Fig. 1 ▸).

### 3.2. Quality measures for protocol development   

Large macromolecular complexes tend to be structurally unstable and often assume several different, simultaneously present conformations. Unsuitable purification methods can induce disassembly and aggregation or small structural changes that may be mis­interpreted as conformational variability. It is therefore essential to use appropriate protein-purification methods to prevent dis­assembly and denaturation during purification and cryoEM sample preparation (Chari *et al.*, 2015 ▸). The small percentages of picked particles in many cryoEM reconstructions suggest that the majority are damaged. In many instances the proportion of intact particles is below 20% [19% for human synaptic GABAA receptor (Zhu *et al.*, 2018 ▸), 15% for human P-glycoprotein (Kim & Chen, 2018 ▸), 11.8% for nucleosome (Takizawa *et al.*, 2018 ▸), 8.9% for human γ-secretase (Bai *et al.*, 2015 ▸) and 5.7% for sodium channel from electric eel (Yan *et al.*, 2017 ▸)]. Frequently, it is not clear whether the macromolecular complex suffered during protein production or sample preparation for cryoEM.

Each step in our protocol for the rapid preparation of yeast FAS for high-resolution structural studies was examined rigorously. Quality criteria included oligomeric state and thermal stability, monitored by size-exclusion chromatography (SEC), and thermal unfolding, monitored by sparse-matrix screening (TSA) (Ericsson *et al.*, 2006 ▸). Both methods are sensitive tools for screening protein preparation conditions. Further, the catalytic activity of FAS served as a measure of overall protein integrity. Specific catalytic activity, determined as the catalytic activity of the probe related to the FAS concentration as judged by SDS–PAGE, proved to be ideal for optimizing the vector-based expression system and assessing progress in the purification protocol. Amongst other things, we found that the C-terminus of subunit β tolerated tagging, while tagging at the C-terminus of subunit α [in the phosphopantetheine transferase (PPT) domain] prevented complex assembly (data not shown). As outlined in Fig. 1 ▸, SEC, TSA and activity assays were used routinely to check the protein quality of each preparation.

**Figure 2:**
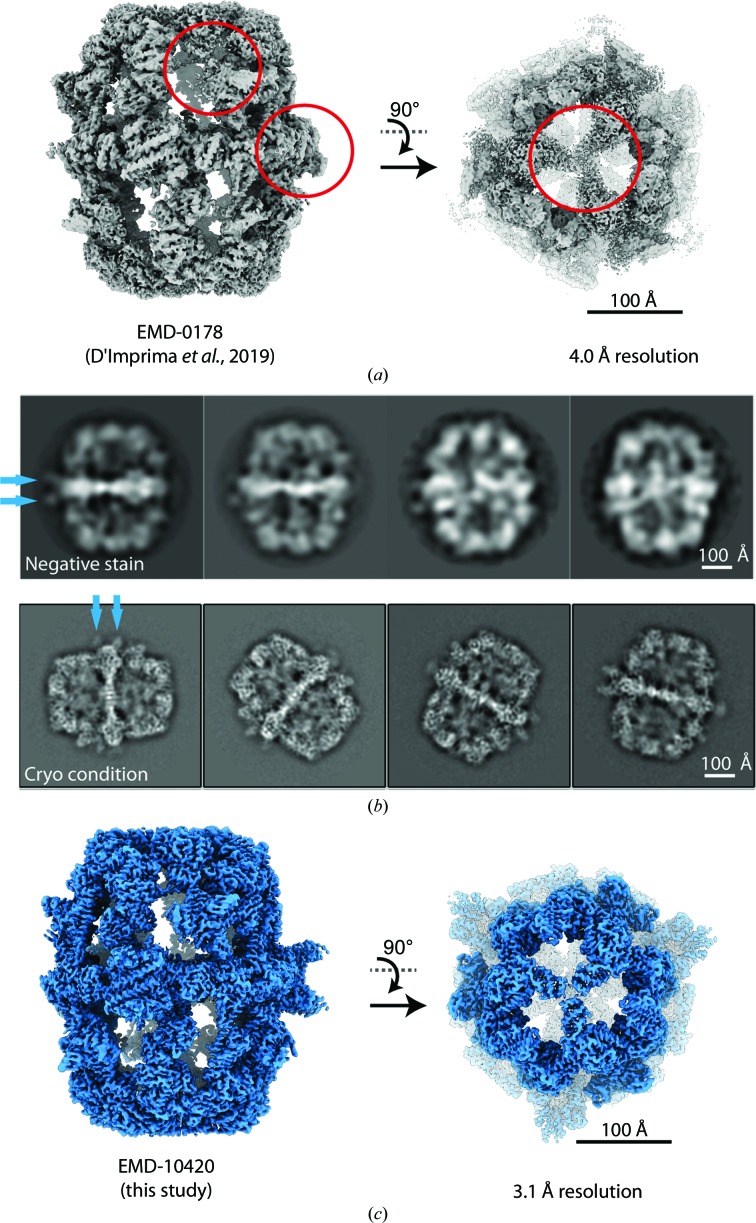
Comparison of FAS preparations. (*a*) The published map (D’Imprima *et al.*, 2019 ▸) lacks the PPT domain and parts of the β-domes are poorly resolved (red circles). (*b*) Data collected using protein prepared by the optimized protocol described here. The 2D class averages show structured PPT domains (blue arrows) and resolved secondary-structure features at the β-domes. (*c*) CryoEM map from 15 000 particles at 3.1 Å resolution.

As another valuable diagnostic of protein stability (Gao *et al.*, 2016 ▸; Thompson *et al.*, 2016 ▸), negative-stain EM identified the FAS PPT domain as a major source of structural heterogeneity. When the FAS complex was purified by SEC and concentrated by centrifugation through a semipermeable membrane, the PPT domain was absent in 2D class averages and 3D classes (D’Imprima *et al.*, 2019 ▸) [Fig. 2 ▸(*a*)]. When the concentration step was omitted, 2D class averages of negatively stained particles consistently showed the PPT domain on the outside of the FAS central wheel. The concentration step proved to be unnecessary when we used a continuous support layer on the EM grids, which reduces the sample concentration required for specimen preparation by at least one order of magnitude (D’Imprima *et al.*, 2019 ▸). The partial unfolding of the PPT domain was only observable by EM, as it escapes quality control by enzymatic activity and protein-stability measurements. The PPT domain is only required for the initial step of post-translational modification of the carrier protein (ACP) domain, without being directly involved in the fatty-acid synthesis cycle, and poor PPT domain quality is therefore not visible in the NADPH consumption assay. Furthermore, the PPT domain is not integrated into the FAS barrel and does not contribute to its thermal and oligomeric stability (Johansson *et al.*, 2009 ▸). CryoEM was performed with the same FAS batch as used for negative-stain EM [Fig. 2 ▸(*b*)]. CryoEM data indicated that avoiding the concentration step not only preserves the PPT domain density, but also those of other poorly resolved domains [Fig. 2 ▸(*c*)], including the trimerization domain and the acetyltransferase (AT) domain, in particular its interface with the enoyl reductase (ER) domain, which are now equally as well defined as the other FAS domains.

### 3.3. CryoEM of stable, intact FAS   

**Figure 3:**
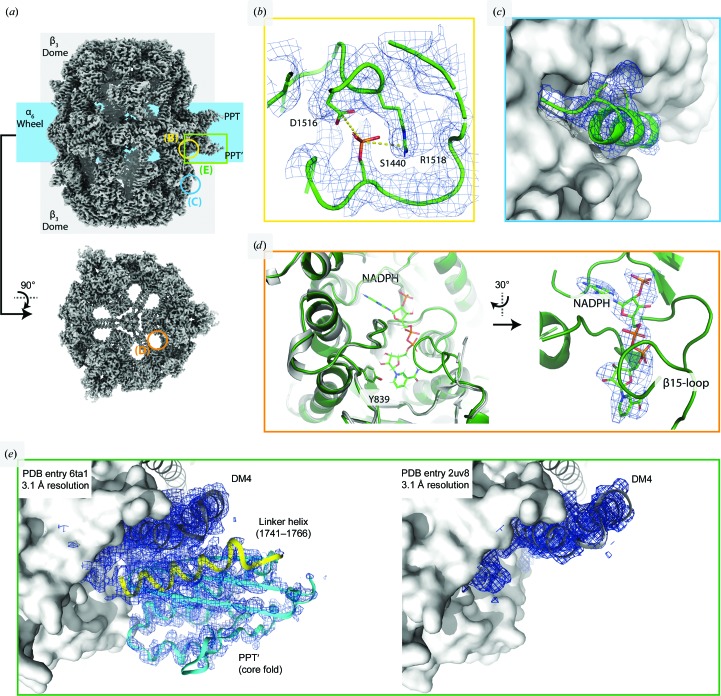
3.1 Å resolution map of FAS. (*a*) Overview of the EM map. The square and circles labelled (B), (C), (D) and (E) indicate the map regions that are enlarged in (*b*), (*c*), (*d*) and (*e*), respectively. (*b*) Density at Ser1440 suggesting phosphorylation. (*c*) Density at residues Cys820 and Cys824 (subunit β) not accounted for by the atomic model. (*d*) NADPH cofactor density in mesh representation, bound to the active site of the KR domain. Left: the KR active site in the apo form as in the X-ray structure (PDB entry 2uv8; gray) superimposed on our cryoEM structure (green). NADPH and the catalytically active Tyr839 are shown in stick representation. (*e*) The PPT domain and the dimerization module DM4, which acts as an adaptor to anchor the PPT domain at the perimeter of the FAS barrel (PPT domain in cyan, DM4 in gray and linker helix in yellow; both densities are shown at 1.0σ). Left: the PPT domain traced in the 3.1 Å resolution cryoEM density. Right: the 3.1 Å resolution X-ray map (data from PDB entry 2uv8; Leibundgut *et al.*, 2007 ▸) shows that DM4 is well resolved, whereas there is no density for the PPT domain or linker helix.

For cryoEM, the FAS sample purified as above was incubated with NADPH and malonyl-CoA prior to plunge-freezing. Although this treatment results in a slight decrease in the thermal protein stability as determined by TSA (Supplementary Fig. S1), it reduces sample heterogeneity by driving the synthesis of bound fatty acids to completion. Protein denaturation at the air–water interface was avoided by applying the sample to a film of graphene on the carbon side of the Quantifoil EM grids. The graphene support was rendered hydrophilic by using 1-pyrenecarboxylic acid as a noncovalent chemical doping agent (D’Imprima *et al.*, 2019 ▸; Section 2). 2D unsupervised class averages revealed that the complex was very stable [Fig. 2 ▸(*b*) and Supplementary Fig. S2]. Three-dimensional reconstruction yielded a map at a global resolution of 3.1 Å [Fig. 3 ▸(*a*)]. In distinction from our previous cryoEM map (EMD-0178; D’Imprima *et al.*, 2019 ▸), the resolution is isotropic (Supplementary Fig. S3) and we were able to build a complete model of yeast FAS (Table 1 ▸).

The new cryoEM map revealed additional density at Ser1440, suggesting that this serine is phosphorylated, as was previously observed in a large-scale phosphorylation analysis in *S. cerevisiae* (Li *et al.*, 2007 ▸) [Fig. 3 ▸(*b*)]. Ser1440 is located in the dimerization module DM4 that holds the PPT domain at the perimeter of the barrel. The phosphate group is embedded in a pocket near Asp1516 and Arg1518. Sequence comparisons revealed high conservation of the Ser1440–Asp1516–Arg1518 motif (Grininger, 2014 ▸). In addition, we found density at Cys820 and Cys824 that is not accounted for by the atomic model [Fig. 3 ▸(*c*)]. The two cysteines are not conserved in fungal FASs, and the density possibly originates from the malonyl group, which binds to cysteine(s) owing to the high malonyl-CoA concentration in solution. In the structure, the NADPH cofactor is bound to the active site of the KR domain [Fig. 3 ▸(*d*)], but not to the ER domain. The active nicotinamide unit is exposed at the inner surface, which contains the acyl-ACP docking sites. Tyr839 sits at the entrance to the binding pocket and is responsible for the protein transfer that neutralizes the hydroxyl anion in the reduction of the carbonyl group by NADPH. This residue was recently mutated to a phenylalanine, turning FAS into a nonreducing, lactone-producing enzyme (Zha *et al.*, 2004 ▸; Gajewski, Buelens *et al.*, 2017 ▸). A comparison with the cofactor-free X-ray structure of baker’s yeast FAS shows the structuring of the β15 loop upon NADPH binding, as observed in the homologous *Thermomyces lanuginosus* type I FAS and type II KR domain (Jenni *et al.*, 2007 ▸) [Fig. 3 ▸(*e*)].

## 4. Discussion   

Within the past five years, cryoEM has developed into a powerful technique for biological structure determination. This is documented by a sharp increase in the number of maps released by the EMDB (from eight in 2002 to 417 in 2012 and 1771 in 2018). Fast and easy access to purified samples is a prerequisite for fully exploiting the technical developments in cryoEM for molecular biology. We have revisited the process of resolving the structure of yeast FAS, a major milestone in early cryoEM and crystallographic studies, and have derived a rapid protocol for determining its complete structure at high resolution.

A number of challenges and pitfalls were revealed during the development of our protocol. In the case of yeast FAS, neither the vector-based expression strategy nor affinity tagging at the C-terminus of subunit β affected the protein quality. However, the PPT domain turned out to be particularly sensitive to partial denaturation. The PPT domain may be prone to denaturation because it is monomeric in the yeast FAS complex (Lomakin *et al.*, 2007 ▸), while it forms trimers as a separate protein (Johansson *et al.*, 2009 ▸). Earlier structures of yeast FAS confirm that the PPT domain is unstable. The PPT domain was not traced in electron densities in the landmark X-ray structures at 3.1–4 Å resolution (Jenni *et al.*, 2007 ▸; Leibundgut *et al.*, 2007 ▸; Lomakin *et al.*, 2007 ▸; Johansson *et al.*, 2008 ▸) [Fig. 3 ▸(*e*)] or in cryoEM maps at 3–4 Å resolution (Lou *et al.*, 2019 ▸; D’Imprima *et al.*, 2019 ▸). We conclude that the PPT domain denatures easily during protein purification, crystallization or cryoEM grid preparation. It is likely that the PPT domain partly unfolds when the protein is concentrated at the solid–liquid interface of the semipermeable membrane (Rabe *et al.*, 2011 ▸). Changes in protein structure resulting from adsorption to solid surfaces are well documented (Tunc *et al.*, 2005 ▸; Norde, 1986 ▸; Höök *et al.*, 1998 ▸; Maste *et al.*, 1997 ▸), ranging from protein denaturation at membranes for water purification (Lee *et al.*, 2016 ▸) to modified behavior of key drug candidates such as amyloid peptides (Zhou *et al.*, 2013 ▸). Strikingly, yeast FAS does not denature upon adsorption to a graphene support film on EM grids, whereas it does denature by interaction with semipermeable membranes or at the air–water interface. Whether and how adsorption to solid surfaces induces protein damage and impairs structure determination at atomic resolution of conformationally weak or unstable proteins will require further investigation.

In conclusion, we present a rapid pipeline for the preparation of the 2.6 MDa yeast FAS with high quality. Together with a companion protocol (D’Imprima *et al.*, 2019 ▸), structural analysis of yeast FAS at ∼3 Å resolution by cryoEM is achievable within a day. While the presented pipeline is unlikely to be directly applicable to other protein complexes, the approach of monitoring and optimizing the individual steps of a purification procedure may serve as a blueprint for other macromolecular assemblies.

## Supplementary Material

EMDB reference: yeast FAS, EMD-10420

PDB reference: yeast FAS, 6ta1

Supplementary Figures. DOI: 10.1107/S2052252519017366/eh5006sup1.pdf

## Figures and Tables

**Table 1:** Statistics of 3D reconstruction and model refinement

Data collection	
Electron microscope	Titan Krios
Electron detector	Falcon III
Voltage (kV)	300
Defocus range (µm)	0.5–2.1
Pixel size (Å)	0.833
Electron dose (e^−^ Å^−2^)	32
Images	792
3D reconstruction	
Final particles	15320
Applied symmetry	*D*3
Resolution (Å)	3.1
*B* factor (Å^2^)	−72
Model composition	
Peptide chains	2
Residues	3780
Cofactors	FMN, NADPH
Ramachandran plot	
Favored (%)	94.27
Outliers (%)	0.13
Validation	
*MolProbity* score	1.96
Rotamer favored (%)	94.01
Rotamer outliers (%)	1.49
