# Supplementary material for: The resolution revolution in cryoEM requires high-quality sample preparation: a rapid pipeline to a high-resolution map of yeast fatty acid synthase
Source: IUCrJ. 2020 Jan 25;7(Pt 2):220–7. doi: 10.1107/S2052252519017366 (PMC7055384; doi:10.1107/S2052252519017366)
Supplement: Supplementary file 1 [file m-07-00220-sup1.pdf]

IUCrJ

Volume 7 (2020)

Supporting information for article:

The resolution revolution in cryoEM requires high-quality sample preparation: a rapid pipeline to a high-resolution map of yeast FAS

Mirko Joppe, Edoardo D'Imprima, Nina Salustros, Karthik S. Paithankar, Janet Vonck, Martin Grininger and Werner Kühlbrandt

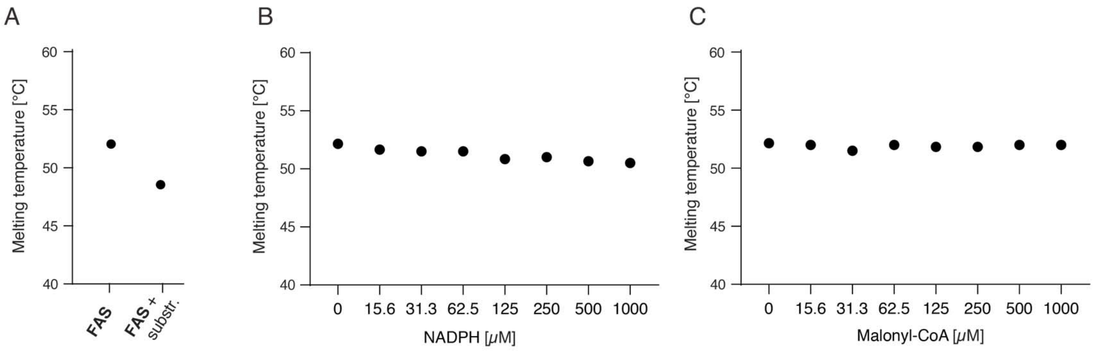

Figure S1 Effect of NADPH and malonyl-CoA on the thermal stability of yeast FAS. The thermal stability of yeast FAS in buffer P1 (100 mM sodium phosphate pH = 6.5) was determined by TSA on one biological sample in three technical replicates. The errors were smaller than 0.5 °C. (A) Thermal stability of yeast FAS before (FAS) and after addition of 1 mM NADPH and 1 mM Mal-CoA (FAS + substr.). (B/C). Thermal stability of yeast FAS at different concentrations of substrates NADPH and malonyl-CoA.

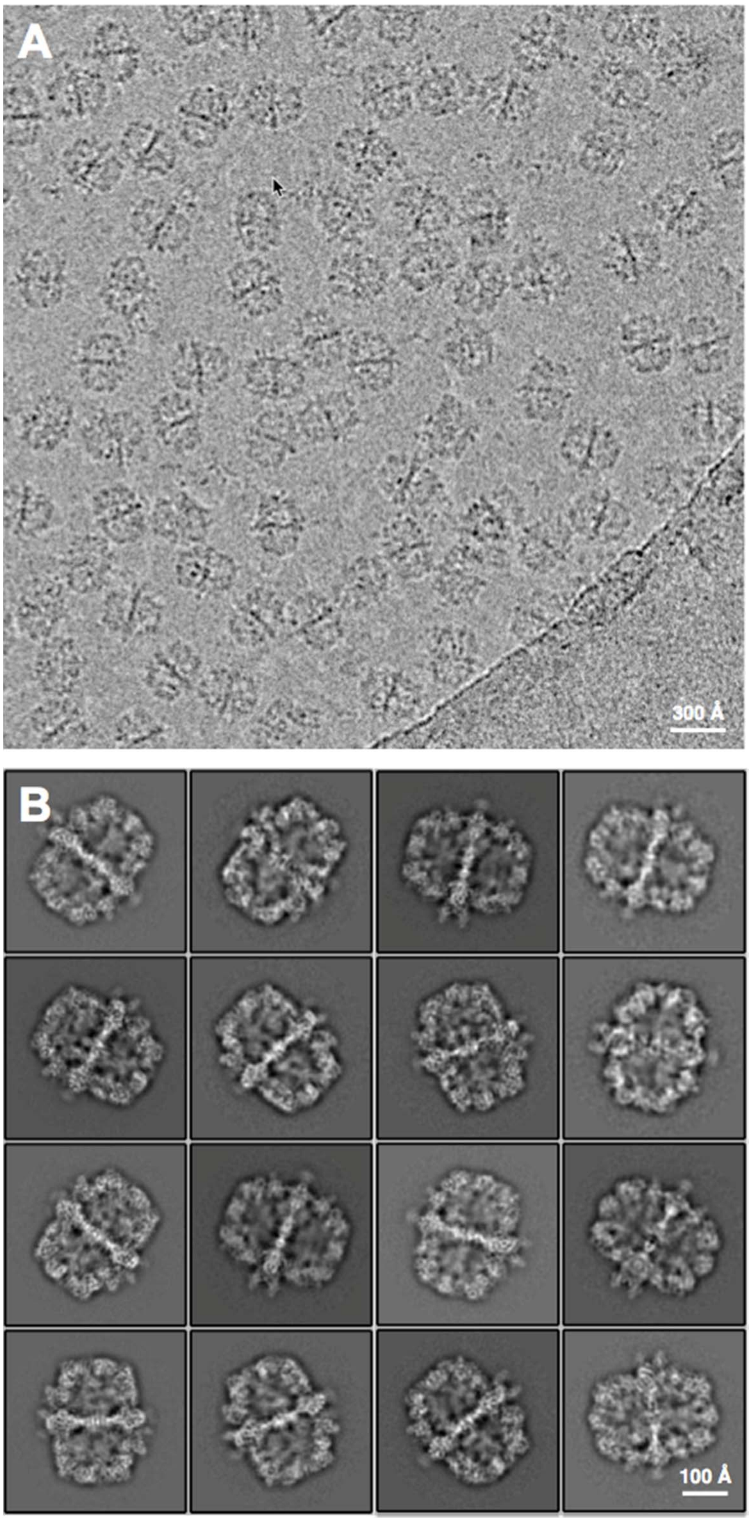

Figure S2 CryoEM of yeast FAS. A: Typical electron micrograph recorded at 1.8 μm defocus shows evenly dispersed particles. B: Two-dimensional class averages display secondary structure features and consistent density for the PPT.

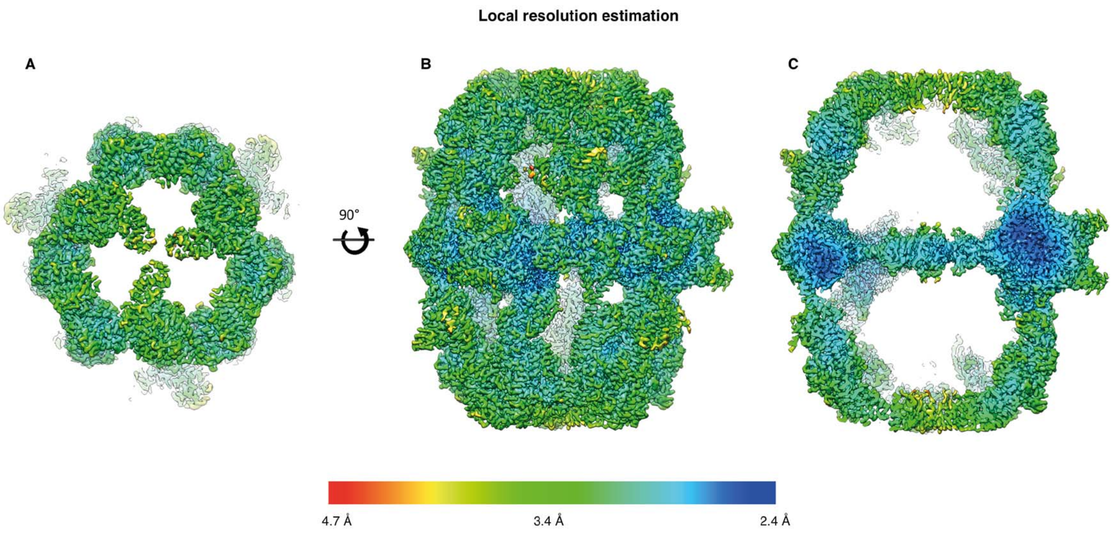

Figure S3 CryoEM reconstruction of yeast FAS colored by local resolution. Yeast FAS is shown in top view (A), side view (B) and as cross-section along the threefold axis (C).
